# Things fall apart

**DOI:** 10.1017/ehs.2022.60

**Published:** 2022-12-21

**Authors:** Ruth Mace

**Affiliations:** Department of Anthropology, University College London, 14 Taviton St, London, United Kingdom of Great Britain and Northern Ireland

## Abstract

**Figure:**
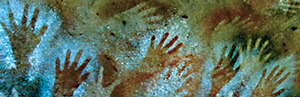

One of the most interesting emails I received this year was from Masanori Kakaota, a philosophy researcher at Hiroshima University, asking if I was the granddaughter of the moral philosopher turned psychologist C. A. Mace (1894–1971), to which the answer was yes. Rather implausibly, it seems that a somewhat forgotten collection of what appears to be the contents of my grandfather's study was purchased by the state of Japan (a country he never visited to my knowledge). I am delighted to learn that a collection of his published and unpublished work is in a university library, although the reason it went to Hiroshima University is not known to any extant members of my family nor indeed to Hiroshima University. However, one point of interest Masanori has in my grandfather is his pacifism. He found in the Hiroshima collection his first academic writing – an unpublished speech on conscience given when he was a student at Queen's College Cambridge in 1916, shortly before his arrest for disobeying orders to fight (his appeal on grounds of conscientious objection was rejected and he was sentenced to prison with hard labour). This email correspondence alerted me to the college magazine my grandfather was editing at the time. Apart from the occasional comment that there were no longer enough rowers to fill the college eight, the rest of the magazine had become basically a list of obituaries of all his fellow students and teachers who had been killed in the war. Most were so young there was little to be said about their short lives other than that they had fought bravely. I am reminded that none of his three sisters ever married, as, after World War I, the shortage of men in their age group was so extreme. In this foray into my family history, I could not help but draw parallels between what happened to his generation and what is happening now in Ukraine, where tens of thousands are dying in not dissimilar circumstances. Of all the disasters occurring this year, a war on the edge of Europe is the most unexpected.

Our woes are trivial compared with those freezing, imprisoned or dying in war. Nonetheless, here in the UK there is a growing feeling that things are falling apart a bit. As I write, we are facing a winter of strikes in most public services, including universities. However, you will be pleased to hear that *Evolutionary Human Sciences* is still going strong. Our submission rate survived the arrival of publication charges (from which, as I keep reminding you, most of you are exempt, largely owing to the very extensive range of ‘transformative’ agreements that Cambridge University Press has negotiated with a great many universities around the world). We do not yet have an ‘impact factor’. For those of you who hate impact factors, *EHS* is an example you can cite of a journal that is ticking along just fine without one. For those of you who like or need impact factors, we hope to get one soon. To those of you who have found the time, grant money and inspiration to write great scientific papers for us, and to review or edit them, I am, as ever, very grateful. Long may it last.

At UCL Anthropology we have decided to respond to the gloomy economic mood by inviting you all to a great big party: the 2023 meeting of the European Human Behaviour and Evolution Association (EHBEA2023), that we will host here in London from 18 to 21 April. I actually hosted a similar meeting at UCL for another society (Human Behavior and Evolution Society, HBES) way back in 2001 (before EHBEA existed), and it took me 20 years to recover enough to consider doing it again. We are using the same lecture theatres, which I fear may not have been refurbished since the last time. However, we will make up for a lack of investment in UK universities with a cool conference at a cool location – London may be getting a bit shabby but has plenty to offer as ever. Thanks to generous sponsorship, especially from our friends from across the pond at the WennerGren foundation, which has a long history of occasionally sponsoring EHBEA meetings, and also thanks to UCL for not charging us for room hire, the registration fee is very reasonable. So if you are a member of EHBEA, or if you join, you can submit your abstracts now through the CUP portal https://ehbea2023.wixsite.com/ehbea-2023

In our opening plenary Adam Rutherford will talk about the history of eugenics, a topic made even more relevant given that we have a rather dodgy history at UCL in that area. Our other plenaries span evolutionary ecology and anthropology, gene-culture co-evolution and evolutionary psychology. We are holding a lunchtime meeting at the conference on how to be an effective ally to those in under-represented groups, courtesy of the REED network https://www.britishecologicalsociety.org/membership-community/reednetwork/. Immediately prior to the main EHBEA meeting, we are hosting a satellite meeting on researching sensitive topics. Many of our interests focus on sensitive or controversial practices that are difficult to study, especially in a quantitative way. However, we cannot leave these topics hidden outside the remit of scientific and anthropological investigation, especially if they concern practices that can inflict misery and harm. The workshop will have talks on both the ethics and practicalities of how to get quantitative data on cultural practices or controversial beliefs that are not easy to measure directly. If you would like to attend that workshop, you need to arrive in London a couple of days early (17–18 April – website for registration coming soon). So, we look forward to seeing as many of you as possible (up to a hard limit of 300) in London in person in April. Please come, and please come by train or boat or bicycle if you can, as we do not want to fry the planet (we are still connected to mainland Europe by the Eurostar, just).

One pronounced trend in our field is revision in how we use statistics. Causal inference turns out to be more complicated than we thought. I am not dogmatic on methods. I tend to take the long view that statistical fashions are always changing, although it is a bit alarming to be told that some of our favourite methods are wrong. Change in knowledge is a good thing, and we may need to catch up with some other disciplines in this regard. We are now calling for papers for a special collection on causal inference to help us with this, so do get in touch with Charles Efferson (charles.efferson@unil.ch), who is the leading editor, if you would like to contribute a paper to this collection https://www.cambridge.org/core/journals/evolutionary-human-sciences/announcements/call-for-papers/special-themed-issue-causal-inference

(Research papers, Perspectives and Methods papers are all welcome).

So my Christmas card to you, courtesy of Dan Major-Smith, (whose stage one registered report for said volume is available here https://osf.io/z5gcm/files/osfstorage) is

**Figure:**
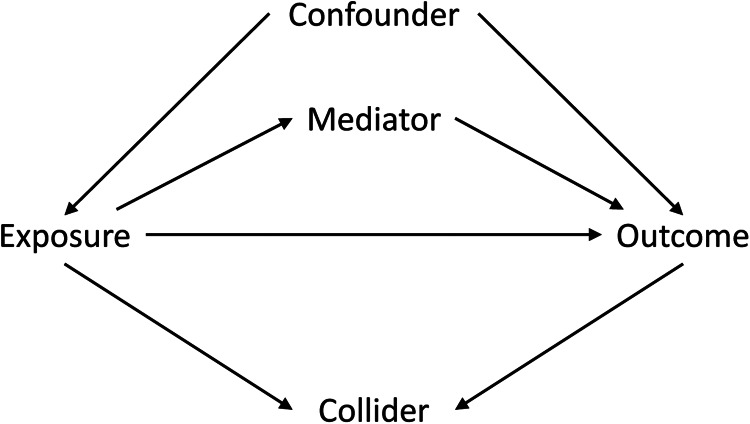

I hope you are having a very happy Christmas break and wish you a truly peaceful 2023

